# Supplementary material for: Quantifying Information without Entropy: Identifying Intermittent Disturbances in Dynamical Systems
Source: Entropy (Basel). 2020 Oct 23;22(11):1199. doi: 10.3390/e22111199 (PMC7712588; doi:10.3390/e22111199)
Supplement: Supplementary file 1 [file entropy-22-01199-s001.zip › entropy-958565-supplementary.html]

IIF\_stft 

## Contents

- Information Impulse Function (IIF) Using Short-time Fourier Transform:

```
function [iifR,T,iifL,F,rho] = IIF_stft(fs,signal,p)
```

## Information Impulse Function (IIF) Using Short-time Fourier Transform:

The IIF detects and time-localize small disturbances in the response data of a nonlinear system. Relative information content is measured without using Boltzmann’s equation by modeling signal transmission as a series of dissipative steps. The resulting informational structure can be used to detect disturbances in the response signal. This implementation of the IIF uses the short-time Fourier transform (STFT), and is deal for signals of total length between 3000 and 5000 points.

```
%%%%%%%%%%%%%%%%%%%%%%%%%%%%%%%%%%%%%%%%%%%%%%%%%%%%%%%%%%%%%%%%%%%%%%%%%%%
%                               INPUTS
%%%%%%%%%%%%%%%%%%%%%%%%%%%%%%%%%%%%%%%%%%%%%%%%%%%%%%%%%%%%%%%%%%%%%%%%%%%
%    fs      sample rate in Hz
%    signal  one dimensional array of response values
%    p       exponent defining the STFT window size (window_size = 2^p)
%%%%%%%%%%%%%%%%%%%%%%%%%%%%%%%%%%%%%%%%%%%%%%%%%%%%%%%%%%%%%%%%%%%%%%%%%%%
%                               OUTPUTS
%%%%%%%%%%%%%%%%%%%%%%%%%%%%%%%%%%%%%%%%%%%%%%%%%%%%%%%%%%%%%%%%%%%%%%%%%%%
%   iifR     one dimensional array of iifR values
%   T        one dimensional array of time values. Length matches the iifR
%   iifL     one dimensional array of iifL values
%   F        one dimensional array of frequency values. Length matches the
%                iifL
%   rho      rank of the decomposition
%%%%%%%%%%%%%%%%%%%%%%%%%%%%%%%%%%%%%%%%%%%%%%%%%%%%%%%%%%%%%%%%%%%%%%%%%%%
%                             REFERENCE
%%%%%%%%%%%%%%%%%%%%%%%%%%%%%%%%%%%%%%%%%%%%%%%%%%%%%%%%%%%%%%%%%%%%%%%%%%%
% Additional details, derivations and case studies for the IIF can be found
% the full manuscript published in Entropy:
% A. Montoya,E. Habtour,F. Moreu, "Quantifying information without entropy:
% identifying intermittent disturbances in dynamical systems" (2020)
%%%%%%%%%%%%%%%%%%%%%%%%%%%%%%%%%%%%%%%%%%%%%%%%%%%%%%%%%%%%%%%%%%%%%%%%%%%
%                             DISCLAIMER
%%%%%%%%%%%%%%%%%%%%%%%%%%%%%%%%%%%%%%%%%%%%%%%%%%%%%%%%%%%%%%%%%%%%%%%%%%%
% This work is part of the Advanced Simulation and Computing (ASC)
% initiative at Sandia National Laboratories. Sandia National Laboratories
% is a multi-mission laboratory managed and operated by National Technology
% and Engineering Solutions of Sandia, LLC, a wholly owned subsidiary of
% HoneywellInternational Inc., for the U.S. Department of Energy’s National
% Nuclear Security Administration under contract DE-NA0003525.
%%%%%%%%%%%%%%%%%%%%%%%%%%%%%%%%%%%%%%%%%%%%%%%%%%%%%%%%%%%%%%%%%%%%%%%%%%%
%                           SOFTWARE ABOUT
%%%%%%%%%%%%%%%%%%%%%%%%%%%%%%%%%%%%%%%%%%%%%%%%%%%%%%%%%%%%%%%%%%%%%%%%%%%
% IIF_stft,  Version 1.0 | November, 2019
%
% Written for use with
%   MATLAB Version: 9.7.0.1190202 (R2019b)
%   Signal Processing Toolbox Version 8.3 (R2019b)
%%%%%%%%%%%%%%%%%%%%%%%%%%%%%%%%%%%%%%%%%%%%%%%%%%%%%%%%%%%%%%%%%%%%%%%%%%%
%                               Author
%%%%%%%%%%%%%%%%%%%%%%%%%%%%%%%%%%%%%%%%%%%%%%%%%%%%%%%%%%%%%%%%%%%%%%%%%%%
% A.C. Montoya
% Sandia National Laboratories
% Albuquerque, NM 87185, USA
% contact: acmont@sandia.gov
```

```
% Adjust inputs if needed
if ~iscolumn(signal)
    signal = signal';
end

% Decompose the signal
win  = hann(2^p,'periodic');
nfft = length(win)*2;
[W,F,T] = stft(signal,fs,'Window',win,'FFTLength',nfft);

% Determine the rank
rho = rank(W);

% Run the IIF
[iifR,iifL] = IIF_main(W,rho);
```

```
end

% IIF Main Engine
function [dR,dL] = IIF_main(W,rho)
% Calculate the SVD, ignore the singular values
[U0,~,V0] = svd(W);

% The sum of these is the diagonal of the identity matrix
% (V0*V0', and U0*U0',respectively)
U2 = real(U0).^2 + imag(U0).^2;
V2 = real(V0).^2 + imag(V0).^2;

% rank spacing
ds = 1/(rho*(rho+1));

% compute work(rank) and area under the curve up to rank rho:
dL = sum(cumsum(U2(:,1:rho),2),2).*ds;
dR = sum(cumsum(V2(:,1:rho),2),2).*ds;
end
```

Published with MATLAB® R2019b
